# Small Intestinal Digestive Functions and Feed Efficiency Differ in Different Pig Breeds

**DOI:** 10.3390/ani13071172

**Published:** 2023-03-26

**Authors:** Yating Cheng, Sujuan Ding, Md. Abul Kalam Azad, Bo Song, Xiangfeng Kong

**Affiliations:** 1 Key Laboratory of Agro-Ecological Processes in Subtropical Region, Hunan Provincial Key Laboratory of Animal Nutritional Physiology and Metabolic Process, National Engineering Laboratory for Pollution Control and Waste Utilization in Livestock and Poultry Production, Institute of Subtropical Agriculture, Chinese Academy of Sciences, Changsha 410125, China; 2 College of Advanced Agricultural Sciences, University of Chinese Academy of Sciences, Beijing 100008, China

**Keywords:** pig breed, feed efficiency, small intestine, digestive function, nutrients

## Abstract

**Simple Summary:**

The advantages and disadvantages of production performance in pig production, such as growth performance and digestive ability, are always affected by genetic and exogenous factors; in particular, diets are the top consideration, while genetic effects play an important role. Thus, the aim of this study was to investigate the differences between pig breeds (Taoyuan black, Xiangcun black, and Duroc pigs) in the small intestinal digestive functions and feed efficiency. Our results show that the different pig breeds have significantly different growth performance, metabolic levels, and apparent total tract digestibility, mainly resulting from the differences in small intestinal digestive functions, and the changes might be related to age. These findings provide promising guidance for further feed preparation and breed selection for the pig industry.

**Abstract:**

Small intestinal growth and health affect its digestion and absorption ability, while little information exists about the small intestinal morphology and function differences among the different pig breeds. Therefore, 90 healthy 35 days of age Taoyuan black (TB), Xiangcun black (XB), and Duroc (DR) pigs (30 pigs per breed) with similar body weight (BW) of the same breed were reared to 185 days of age to evaluate the potential relationship between feed efficiency and small intestinal morphology and function at 80, 125, and 185 days of age. The results show that the TB and XB pigs had lower initial and final BW, ADG, and ADFI and plasma CHO and LDL-C levels, whereas they had higher plasma LIP levels and jejunal trypsin, invertase, lactase, and maltase activities and higher DM, ADF, Tyr, Arg, and His digestibility at 80 days of age compared with the DR pigs. At 125 days of age, TB and XB pigs had lower apparent total tract digestibility and plasma CHO, HDL-C, LDL-C, and NH_3_ levels; XB pigs had lower DM and NDF digestibility, and TB pigs had higher jejunal lactase and maltase activities. At 185 days of age, TB and XB pigs had lower DM, EE, ADF, and GE digestibility, while having higher plasma ALT and UN levels; TB pigs had higher plasma AST level and jejunal chymase activity. Furthermore, the plasma free amino acid contents, small intestinal VH, and nutrient transporter expression levels differed at different ages. Therefore, the different pig breeds exhibited significantly different growth performance and small intestinal growth, mainly resulting from the differences in digestive enzymes and nutrient transporters in the small intestine.

## 1. Introduction

Feeding cost generally accounts for 60% to 70% of the total pig production cost and is still increasing gradually due to the increasing shortage of feed sources in recent years [1]. Furthermore, the increasing cost of pig production may result in higher pork prices and lower sales, which may cause a vicious circle between the pig industry and consumers, thus restricting the sustainable development of pig industries [2]. The growing global population and lifestyle changes have increased the demand for pork production, especially in China [3]. Thus, improving the feed efficiency of pigs may play a critical role in solving this problem, and extensive research has been carried out on the influencing factors and feed strategy related to feed efficiency for pig production. For example, Ding et al. [4] reported that the feed efficiency of animals is associated with hormonal and digestive gland secretion during feeding. In addition, Aliakbari et al. [5] found that the higher feed efficiency in pigs was related to the higher gut microbial diversity, which resulted in better gut health and resilience to feed changes. McCormack et al. [6] reported that pigs with higher feed efficiency had a higher relative abundance of potentially beneficial bacteria (e.g., *Clostridiales* and *Bacteroidetes*) and a lower relative abundance of potentially pathogenic bacteria (such as *Rhodococcus* and *Erysipelotrichaceae*). Collectively, most studies aimed to define the relationship between feed efficiency and body metabolism or intestinal microbiome. However, little information exists about the association between digestive capacity and feed efficiency in pigs.

The small intestine is not only the main site for the nutrient digestion and absorption in animals, but also a major part of nutrient sensing to regulate feed intake and digestive function [7]. Thus, the small intestinal digestive capacity is important in regulating feed digestion and utilization. Meyer et al. [8] reported that small intestinal growth indicators (including mass, length, and tissue density) were positively correlated with residual feed intake and associated with the variation in the efficiency of nutrient utilization in cattle. Furthermore, Montanholi et al. [9] found that the higher feed efficiency is related to better small intestinal histomorphometric parameters in bovines, such as cellularity, cell size, and crypts. However, little information exists about the correlation between small intestinal function and digestive capacity in pigs. Therefore, determining the potential correlation between feed efficiency and the small intestinal function will offer promising perspectives for selecting higher feed efficiency breeds based on the small intestinal growth in pigs.

Different pig breeds may have different feed preferences and feed efficiency due to their phenotype traits, environmental adaptation, and availability of feed resources. Duroc (DR) pig is symbolized by a higher growth rate, lean meat, and feed efficiency and is widely used in breeding programs as the terminal sires for crossed commercial pigs. Chinese domestic pigs, including Xiangcun black (XB) and Taoyuan black (TB) pigs, are symbolized by higher stress resistance and roughage feeding tolerance and lower growth rates and lean meat [10]. Homma et al. [11] reported that feed efficiency and resilience traits were heritable, and the influence of genetic factors on them could be the same among the Large White, Landrace, and DR pigs. Bergamaschi et al. [12] found that the feed efficiency among DR, Landrace, and Large White pigs was correlated with the intestinal microbiome composition in these pig breeds. Therefore, we hypothesized that the differences in the small intestinal digestive capacity of different pig breeds might result in differential feed efficiency between Chinese domestic and foreign commercial pigs. Therefore, the present study aimed to determine the association between feed efficiency, tract digestibility, intestinal morphology, digestive enzyme activity, and nutrient transporters among TB, XB, and DR pigs at different ages.

## 2. Materials and Methods

### 2.1. Animals Feeding and Sample Collection

Ninety healthy 35-day-old TB, XB, and DR pigs (30 pigs per breed) with similar body weight (BW; TB = 11.2 ± 1.71 kg, XB = 10.74 ± 1.45 kg, and DR = 11.44 ± 1.5 kg) of the same breed were selected and housed in individual pens (TB and XB piglets were half male and half female, and all the DR piglets were male). Chinese domestic pigs (TB and XB pigs) were obtained from Xiangcun High-Tech Agricultural Co., Ltd. (Loudi, China), and DR pigs were obtained from Tianxin Breeding Share Co., Ltd. (Changsha, China). All pigs were vaccinated according to the vaccination program of commercial pig farms prior to the trial; pigs did not receive any vaccinations during the trial. The pigs were fed pre-nursery and late-nursery diets from 35 to 80 days of age, growing diets from 81 to 125 days of age, and finishing diets from 126 to 185 days of age. Each pen was equipped with a water nipple and a single-hole feeder to access water and feed at all times. The composition and nutrient levels of diets for different breeds of pigs (Table 1) met the Chinese local swine nutrient requirements (NY/T 65-2004), and the premixes met the National Research Council (NRC, 2012) diet requirements [13,14].

Within seven days before 80, 125, and 185 days of age, 0.1% exogenous indicator titanium dioxide was added to the feed as described previously by Jagger et al. [15], and the fresh fecal samples from each pig (10 pigs per breed) were collected during the final three days of each phase. The fecal samples were mixed after adding 10% dilute sulfuric acid for nitrogen fixation and then stored at −20 °C for nutrient digestibility analysis. Approximately 300 g feed samples were collected and stored at −20 °C to determine the nutritional components contents.

At 80, 125, and 185 days of age, 12 h after the last feeding, 10 pigs from each breed with similar BW were randomly selected, weighed, and then euthanized under commercial conditions via electrical stunning (120 V, 200 Hz). All organs were removed and separated for sampling. The intestinal segments were immediately opened lengthwise following the mesentery line, flushed with ice-cold saline (154 mM NaCl, 0.1 mM PMSF, pH 7.4), and divided into jejunal and ileal mucosa samples for digestive enzyme activity analysis. The jejunum (10 cm below the duodenum–jejunum junction) and ileum (10 cm above the ileocecal junction) tissue samples were collected, frozen in liquid nitrogen, and immediately stored at −80 °C for gene expression analysis. In addition, 0.5–1 cm jejunal and ileal tissues were collected immediately after euthanization and fixed with 10% neutral buffered formalin until processing for histological analysis.

### 2.2. Growth Performance Analysis

The feed intake was documented daily for each pig, and the initial BW and final BW (12 h fasting) were measured for three phases, including 35–80, 80–125, and 125–185 days of age. The average daily gain (ADG), average daily feed intake (ADFI), and feed/gain ratio (FCR) were calculated.

### 2.3. Apparent Total Tract Digestibility Analysis

The contents of nutritional components were analyzed for dry matter (DM) by oven-drying at 105 °C. The gross energy (GE) was determined by using benzoic acid as the calibration standard in an isothermal auto-colorimeter ((Changsha Kaide Observe And Control Instrument Co., Ltd., Changsha, China). Crude protein content (N × 6.25) was determined by the combustion method using an AA3 flow injection analyzer (SEAL Analytical GmbH, Norderstedt, Germany). The concentration of titanium (Ti) in feed and fecal samples was analyzed using an inductively coupled plasma optical emission spectrometer (Agilent Technologies Inc., Santa Clara, CA, USA). The apparent total tract digestibility (ATTD) of nutritional components was calculated using the following equation [16]: ATTD (%) = 100 – [100 × (concentration of TiO_2_ in feed × concentration of component in feces/ (concentration of TiO_2_ in feces × concentration of component in feed))].

### 2.4. Plasma Biochemical Parameter Analysis

The plasma biochemical parameters (*n* = 10), including alanine aminotransferase (ALT), albumin (ALB), ammonia (NH_3_), amylase (AMS), aspartate aminotransferase (AST), cholinesterase (CHE), cholesterol (CHO), glucose (GLU), high-density lipoprotein-cholesterol (HDL-C), lactate dehydrogenase (LDH), low-density lipoprotein-cholesterol (LDL-C), lipase (LIP), triglyceride (TG), total protein (TP), and urea nitrogen (UN) were analyzed using commercially available biochemical kits (Leadman Biochemistry Technology Company, Beijing, China) on an Automatic Biochemical Analyzer (F. Hoffman-La Roche Ltd., Basel, Switzerland) according to the manufacturer’s instructions.

### 2.5. Plasma Free Amino Acid Content Analysis

The plasma samples (*n* = 10) were obtained by centrifuging at 6500× *g* for 10 min, and 600 μL of supernatant was taken into a new centrifuge tube. Then an equal volume of 8% sulfosalicylic acid solution was mixed and then stored at 4 °C overnight to precipitate proteins. After centrifuging at 6500× *g* for 10 min, the supernatant was filtered through a 0.22 μm membrane. Finally, the contents of free amino acids, including 3-methylhistidine (3-MH), alanine (Ala), alpha-aminoadipic acid (α-AAA), alpha-aminobutyric acid (α-ABA), arginine (Arg), aspartic acid (Asp), beta-alanine (β-Ala), citrulline (Cit), cystathionine (Cysthi), cysteine (Cys), ethanolamine (ETA), glutamic acid (Glu), glycine (Gly), histidine (His), hydroxyproline (HYP), isoleucine (Ile), leucine (Leu), L-ornithine (Orn), lysine (Lys), methionine (Met), phenylalanine (Phe), phosphoserine (P-Ser), proline (Pro), sarcosine (Sar), serine (Ser), taurine (Tau), threonine (Thr), tyrosine (Tyr), and valine (Val) were measured using an automatic amino acid analyzer (Hitachi, Tokyo, Japan).

### 2.6. Intestinal Morphology Analysis

The fixed samples of the jejunal and ileal tissues were trimmed, dehydrated, and embedded in paraffin. Slides with 5 μm tissue were stained with hematoxylin–eosin following the manufacturer’s instructions (Wuhan Servicebio Technology Co., Ltd., Wuhan, China). Ten well-oriented villi and crypts were chosen per slide, and a total of 10 slides from each breed were measured. The villus height (VH) and crypt depth (CD) were measured in random fields at 40 × magnification under light microscopy (Olympus, Tokyo, Japan) using the Case viewer image software (Digital Pathology Company, Budapest, Hungary), and then the VH/CD ratio was calculated.

### 2.7. Digestive Enzyme Activity Analysis

Jejunal and ileal mucosa (*n* = 8) were thawed on ice. Mucosa (1 g) was extracted by adding 9 mL ice-cold PBS (1×), vortexed, and then centrifuged at 4 °C and 3000× *g* for 15 min. The levels of amylase, chymase, invertase, lactase, lipase, maltase, and trypsin were determined using commercial kits (Shanghai Huyu, Shanghai, China) following the manufacturer’s instructions. A Multiscan Spectrum Spectrophotometer (Tecan, Infinite M200 Pro, Basel, Switzerland) was used for absorbance values. The enzyme activities were normalized to the total protein concentration quantified by the Pierce BCA Protein Assay Kit (Shanghai Huyu, Shanghai, China) and calculated by the total protein unit.

### 2.8. Nutrient Transporter Gene Expression Analysis

The gene expression levels of the intestinal nutrient transporters were measured by real-time reverse transcription quantitative polymerase chain reaction (RT-qPCR), as previously described by Azad et al. [17]. The total RNA of the jejunal and ileum tissues (*n* = 8) was isolated using TRIzol Reagent (Invitrogen, Shanghai, China) following the manufacturer’s instructions. The concentration of the total RNA was quantified using a Nanodrop 2000 instrument (Thermo Scientific, Waltham, MA, USA), and 500 ng of RNA was reversely transcribed to cDNA using TaqMan Reverse Transcription Reagents (Thermo Fisher Scientific, MA, USA). The qPCR assays were conducted using the Premix Ex Taq Kit (TaKaRa Biotechnology Co., Ltd., Dalian, China). The PCR conditions were as follows: initial denaturation at 95 °C for 30 s, followed by 40 cycles of denaturation at 95 °C for 5 s and annealing at 60 °C for 30 s, and final extension at 72 °C for 30 s. The specific primer sequences for pigs in the GenBank database in NCBI are presented in Table 2. The relative gene expression was calculated using the 2^−ΔΔCt^ method, as described by Wagner et al. [18]. For each sample, the amplification of the glyceraldehyde-3-phosphate dehydrogenase (GAPDH) gene was used to normalize the expression of the target gene.

### 2.9. Statistical Analysis

The data were analyzed using the SPSS 22.0 software (IBM Corporation, Chicago, IL, USA) to perform the one-way analysis of variance (ANOVA). The comparison analysis of different treatment groups was performed using Tukey’s post-hoc test. The pen was considered an experimental unit (*n* = 10) to analyze the growth performance, organ index, ATTD, and intestinal morphology. For gene expression and digestive enzyme activity data, the individual animal served as the experimental unit (*n* = 8). Data are presented as means with their standard error of the mean (SEM) unless otherwise indicated. Differences were considered statistically significant when *p* < 0.05.

## 3. Results

### 3.1. Growth Performance and Feed Efficiency of Three Pig Breeds at Different Ages

The growth performance and feed efficiency of the three pig breeds are presented in Table 3. At 35–80, 80–125, and 125–185 days of age, the initial and final BW, ADG, and ADFI of the TB and XB pigs were lower (*p* < 0.05) than those of the DR pigs. In addition, the FCR of the XB pigs was lower (*p* < 0.05) than that of the DR and TB pigs at 35–80 days of age.

### 3.2. Plasma Biochemical Parameters of Three Pig Breeds at Different Ages

The plasma biochemical parameters of the three pig breeds are presented in Table 4. At 80 days of age, plasma CHO and LDL levels were decreased (*p* < 0.05) while LIP level was increased (*p* < 0.05) in the TB and XB pigs compared with the DR pigs. In addition, plasma AST level was increased (*p* < 0.05) in the TB pigs compared with the DR pigs. At 125 days of age, plasma CHO, HDL-C, LDL-C, and NH_3_ levels were decreased while LIP level was increased in the TB and XB pigs compared with the DR pigs (*p* < 0.05). At 185 days of age, plasma ALT and UN levels in the TB and XB pigs and AST and TG levels in the TB pigs were increased (*p* < 0.05) compared with the DR pigs. In addition, while plasma LDH, NH_3_, and LIP levels in the XB and DR pigs were decreased (*p* < 0.05) than the TB pigs.

### 3.3. Plasma Free Amino Acids Composition of Three Pig Breeds at Different Ages

The plasma free amino acids composition of the three pig breeds at 80 days of age is presented in Table 5. Plasma α-AAA, Arg, Car, and Hylys levels were decreased (*p* < 0.05) while Phe, Tyr, and Val levels were increased (*p* < 0.05) in the XB and DR pigs than the TB pigs. Plasma Leu level was increased (*p* < 0.05) in the DR pigs than the TB pigs. Compared with the XB pigs, plasma Lys, Sar, and Ser levels of the TB and DR pigs were decreased (*p* < 0.05), while the ETA level of the DR pigs was increased (*p* < 0.05). Moreover, α-ABA, His, Thr, and Tyr levels were decreased (*p* < 0.05) and Ala, Asp, Gly, Glu, and Pro levels were increased (*p* < 0.05) in the TB and XB pigs compared with the DR pigs.

The composition of plasma free amino acids of the three pig breeds at 125 days of age is presented in Table 6. Compared with the TB pigs, α-AAA and Gly levels of the DR pigs and ETA level of the XB and DR pigs were decreased (*p* < 0.05); Ala level of the XB pigs, β-Ala level of the DR pigs, and P-ser level of the TB and DR pigs were increased (*p* < 0.05). Glu level of the TB and DR pigs was decreased (*p* < 0.05), while P-ser level of the TB and DR pigs was increased (*p* < 0.05) than the XB pigs. Tau level was decreased (*p* < 0.05) in the TB and XB pigs than the DR pigs.

The composition of plasma free amino acids of the three pig breeds at 185 days of age is presented in Table 7. Arg, β-Ala, and Phe levels of the XB and DR pigs and 3-MH level of the DR pigs were decreased (*p* < 0.05) than the TB pigs; Met and Ser levels in the DR pigs and Tyr level of the TB and DR pigs were increased (*p* < 0.05) than the XB pigs; α-ABA, Ala, ETA, Gly, Hypro, Pro, Sar, and Val levels of the TB and XB pigs were decreased (*p* < 0.05), while α-ABA level of the TB and XB pigs was increased (*p* < 0.05) than the DR pigs.

### 3.4. Intestinal Morphology of Three Pig Breeds at Different Ages

Intestinal morphology analysis of the three pig breeds at different ages is shown in Table 8 and Table 9. In the jejunum, the XB pigs had increased (*p* < 0.05) VH at 80 days of age, while TB and XB pigs had decreased (*p* < 0.05) VH at 185 days of age than the DR pigs (Table 8). In the ileum, the TB and XB pigs had decreased (*p* < 0.05) CD than the DR pigs at 80 days of age. Compared with the TB pigs, DR pigs had decreased (*p* < 0.05) ileal VH at 125 days of age, whereas XB and DR pigs had increased (*p* < 0.05) ileal VH at 185 days of age (Table 9).

### 3.5. Intestinal Digestive Enzyme Activities of Three Pig Breeds at Different Ages

Jejunal digestive enzyme activities of the three pig breeds at different ages are presented in Table 10. At 80 days of age, invertase, lactase, maltase, and trypsin activities were increased (*p* < 0.05) in the TB and XB pigs than the DR pigs. Moreover, amylase activity was increased (*p* < 0.05) in the XB and DR pigs than the TB pigs, while lipase activity was increased (*p* < 0.05) in the XB pigs compared with the TB and DR pigs. At 125 days of age, invertase, lactase, and maltase activities were increased (*p* < 0.05) in the TB pigs than the DR pigs; amylase and lipase activities in the XB and DR pigs were increased (*p* < 0.05), while lactase and maltase activities in the XB and DR pigs were decreased (*p* < 0.05) than the TB pigs. At 185 days of age, trypsin, lactase, lipase, and chymase activities in the TB pigs were increased (*p* < 0.05) than the DR pigs; chymase activity in the XB and DR pigs was decreased (*p* < 0.05) than the TB pigs.

Ileal digestive enzyme activities of the three pig breeds at different ages are presented in Table 11. At 80 days of age, amylase activity was increased (*p* < 0.05) in the XB and DR pigs than the TB pigs. Moreover, trypsin, invertase, lactase, lipase, maltase, and chymase activities were decreased (*p* < 0.05) in the TB and DR pigs than the XB pigs. There was no significant difference in digestive activities at 125 and 185 days of age between the three pig breeds.

### 3.6. Apparent Total Tract Digestibility of Three Pig Breeds at Different Ages

The apparent total tract digestibility (ATTD) of the three pig breeds at 80 days of age is presented in Table 12. DM, acid detergent fiber (ADF), Tyr, Arg, and His digestibility values of the TB and XB pigs and GE and Thr digestibility values of the TB pigs were increased (*p* < 0.05), while ADF, GE, crude protein (CP), Ala, Cys, Gly, Ile, Leu, Phe, Pro, Thr, Tyr, Val, Asp, Glu, Lys, and His digestibility of the TB and XB pigs values were decreased (*p* < 0.05) compared with the DR pigs. In addition, DM and NDF digestibility values of the XB pigs were decreased (*p* < 0.05) at 125 days of age than the DR pigs (Table 13).

The ATTD of the three pig breeds at 185 days of age is presented in Table 14. DM, ether extract (EE), ADF, and GE digestibility values of the TB and XB pigs were decreased (*p* < 0.05) than the DR pigs; CP digestibility values of the XB pigs and Cys digestibility values of the DR pigs were decreased (*p* < 0.05) than the TB pigs. Neutral detergent fiber (NDF), Ala, Gly, Ile, Leu, Met, Phe, Pro, Ser, Thr, Tyr, Val, Asp, Glu, Arg, His, and Lys digestibility values of the TB and DR pigs were increased (*p* < 0.05) than the XB pigs.

### 3.7. Nutrient Transporter Gene Expression of Three Pig Breeds at Different Ages

**Figure 1:**
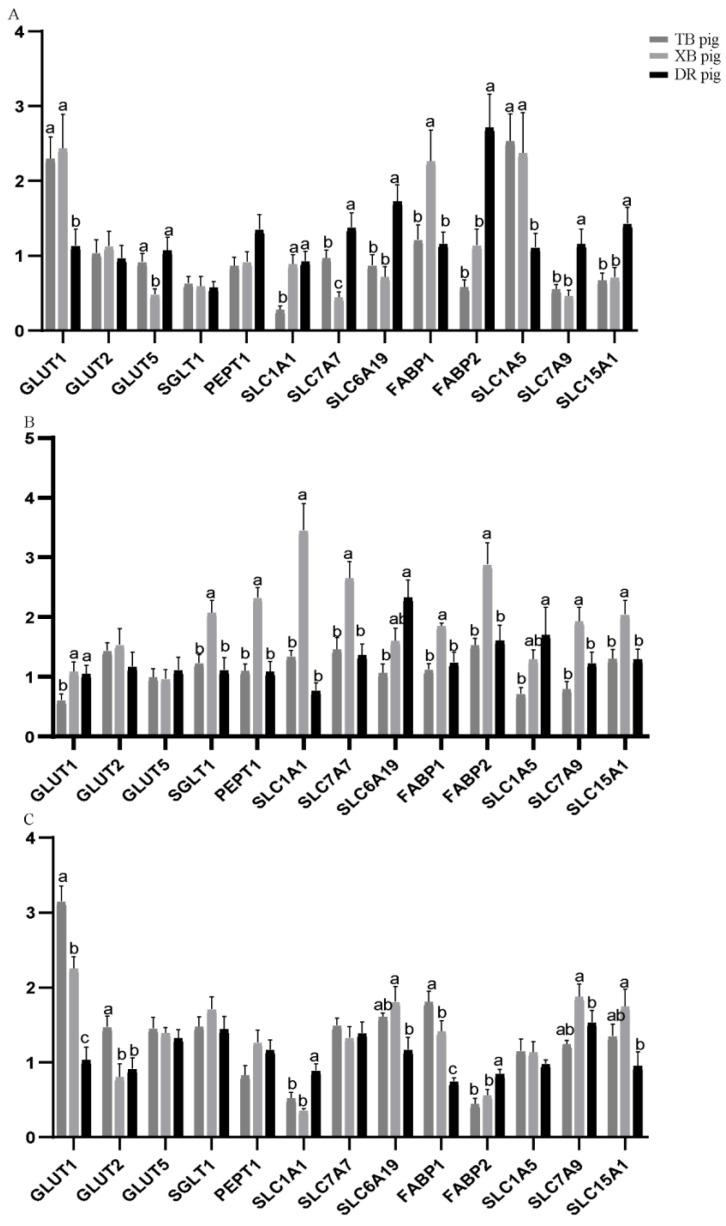
Nutrient transporter gene expression levels in the jejunum of three pig breeds at different ages (*n* = 8): (**A**) 80 days of age; (**B**) 125 days of age; (**C**) 185 days of age. ^a,b,c^ Different letters indicate a significant difference (*p* < 0.05). TB pig, Taoyuan black pig; XB pig, Xiangcun black pig; DR pig, Duroc pig; *FABP*, fatty acid binding protein; *GLUT*, glucose transporter; *PEPT1*, peptide transporter 1; *SGLT1*, sodium–glucose linked transporter 1; *SLC1A1*, solute carrier family 1 member 1; *SLC1A5*, solute carrier family 1 member 5; *SLC6A19*, solute carrier family 6 member 19; *SLC7A7*, solute carrier family 7 member 7; *SLC7A9*, solute carrier family 7 member 9; *SLC15A1*, solute carrier family 15 member 1. The figure below is the same.

Nutrient transporter gene expression levels in the jejunum of the three pig breeds at different ages are shown in Figure 1. At 80 days of age, solute carrier family 7 member 7 (*SLC7A7*), solute carrier family 6 member 19 (*SLC6A19*), fatty acid binding protein 2 (*FABP2*), and solute carrier family 7 member 9 (*SLC7A9*) levels of the TB and XB pigs were lower (*p* < 0.05), while glucose transporter 1 (*GLUT1*) and solute carrier family 1 member 5 (*SLC1A5*) levels of the TB and XB pigs were higher (*p* < 0.05) than the DR pigs; solute carrier family 1 member 1 (*SLC1A1*) level of the DR and XB pigs was higher (*p* < 0.05) than the TB pigs. Furthermore, fatty acid binding protein 1 (*FABP1*) level was lower (*p* < 0.05) and glucose transporter 5 (*GLUT5*) level of the DR and TB pigs was higher (*p* < 0.05) than the XB pigs. At 125 days of age, *SLC6A19* and *SLC1A5* levels of the TB pigs were lower (*p* < 0.05) than the DR pigs; *GLUT1* level of the DR and XB pigs was higher (*p* < 0.05) than the TB pigs, while sodium–glucose linked transporter 1 (*SGLT1*), peptide transporter 1 (*PEPT1*), *SLC1A1*, *SLC7A7*, *FABP1*, *FABP2*, and *SLC7A9* levels of the DR and TB pigs were lower (*p* < 0.05) than the XB pigs. At 185 days of age, *SLC1A1* and *FABP2* levels of the TB and XB pigs were lower (*p* < 0.05) and *SLC6A19* and *SLC7A9* levels of the XB pigs were higher (*p* < 0.05), when compared with the DR pigs; *GLUT1*, glucose transporter 2 (*GLUT2*), and *FABP1* levels of the XB and DR pigs were lower (*p* < 0.05) than the TB pigs.

**Figure 2:**
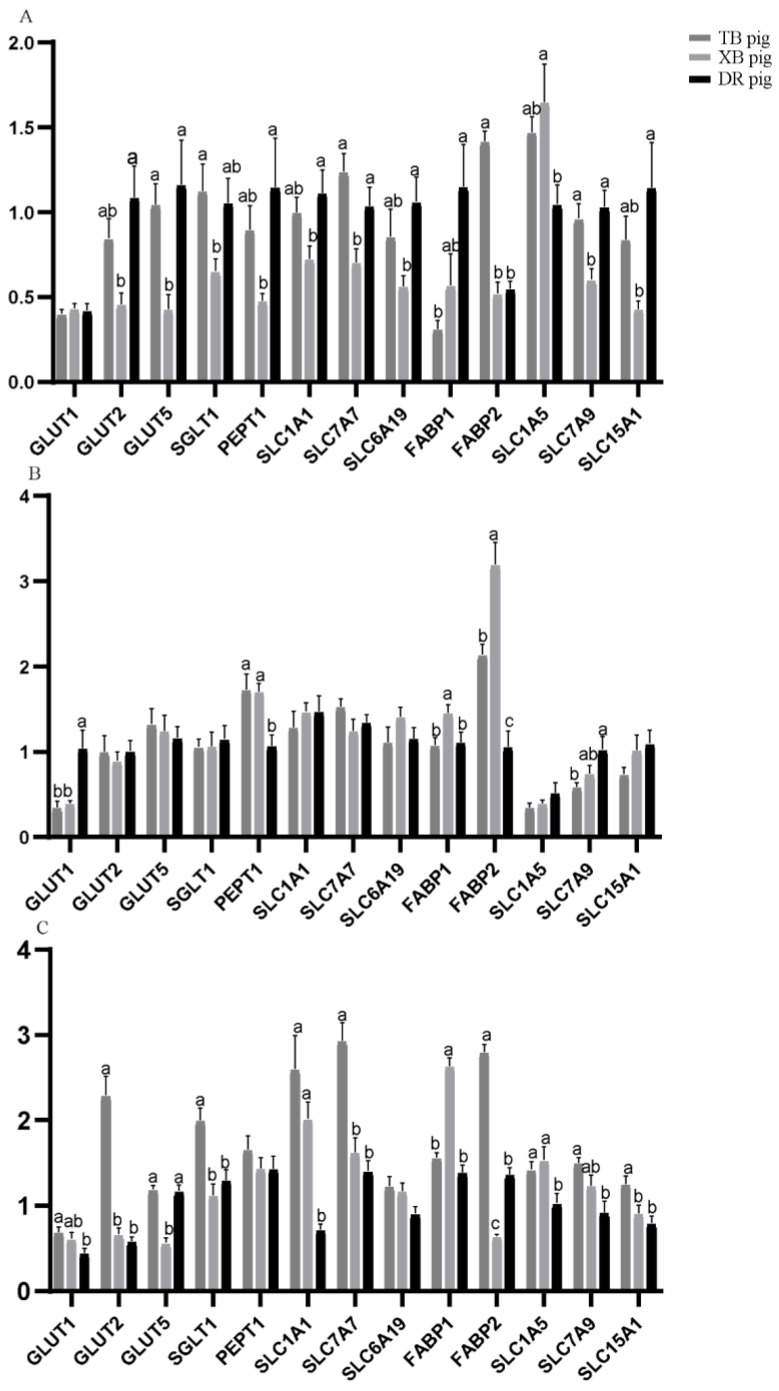
Nutrient transporter gene expression levels in the ileum of three pig breeds at different ages (*n* = 8): (**A**) 80 days of age; (**B**) 125 days of age; (**C**) 185 days of age. ^a,b,c^ Different letters indicate a significant difference (*p* < 0.05).

Nutrient transporter gene expression in the ileum of the three pig breeds at different ages is presented in Figure 2. At 80 days of age, *GLUT2*, *PEPT1*, *SLC1A1*, and *SLC6A19* levels of the XB pigs and *FABP1* level of the TB pigs were lower (*p* < 0.05) than the DR pigs; *SGLT1* and *FABP2* levels were lower (*p* < 0.05), while *SLC1A5* level of the XB pigs was higher (*p* < 0.05) than the TB pigs; *GLUT5*, *SLC7A7*, and *SLC7A9* levels of the DR and TB pigs were higher (*p* < 0.05) than the XB pigs. At 125 days of age, *GLUT1* level of the TB and XB pigs and *SLC7A9* level of the TB pigs were lower (*p* < 0.05) and *PEPT1* level of the TB and XB pigs was higher (*p* < 0.05) than the DR pigs; *FABP1* and *FABP2* levels of the DR and TB pigs were lower (*p* < 0.05) than the XB pigs. At 185 days of age, *SLC1A1* and *SLC1A5* levels of the TB and XB pigs were higher (*p* < 0.05) than the DR pigs; *GLUT1* and *SLC7A9* levels of the DR pigs and *GLUT2*, *SGLT1*, *SLC7A7*, and *FABP2* levels of the DR and XB pigs were lower (*p* < 0.05) than the TB pigs; *GLUT5* level was higher (*p* < 0.05), while *FABP1* level was lower (*p* < 0.05) in the DR and TB pigs than the XB pigs.

## 4. Discussion

Feed efficiency is a crucial economic indicator in pig production, especially for growing pigs. Generally, the assessment of the factors affecting feed efficiency mainly focuses on the microbiome, genetic selection, and diets [19,20]. However, the digestive ability of animals might be highly correlated to the feed efficiency of farm animals. Deru et al. [21] reported that digestibility coefficients were positively correlated with feed efficiency from the genetic point of view and assumed that digestibility could be an interesting trait in breeding schemes. Therefore, the present study aimed to identify the effects of intestinal digestive characteristics on feed efficiency among TB, XB, and DR pigs. Our results showed the differences in small intestinal morphology and function among these three pig breeds at different growth periods.

Feed efficiency in pigs has traditionally been measured by FCR, and a higher FCR represents lower feed efficiency [22]. In the present study, the FCR among three pig breeds had no significant differences; however, the growth performance (including BW, ADG, and ADFI) of the DR pigs was significantly higher than that of the TB and XB pigs. These results suggest that the foreign pig breed has exhibited higher growth performance than the Chinese domestic pig breeds. These findings are consistent with those of Yang et al. [23], who analyzed the whole genomes of foreign and Chinese domestic breeds and found that the higher expression of genes related to the growth and development in the foreign pig breeds was associated with their higher growth performance. Furthermore, the XB pigs had a higher feed efficiency during 35–80 days of age, and the differences disappeared at the other two age stages. Ngoc et al. [24] reported that Landrace × Yorkshire pigs had better feed efficiency than the Mong Cai pigs. This discrepancy reflects the hybridization advantages of the XB pigs, while the disappeared advantages might be related to the insufficient fiber contents in the growing pig’s diet; however, further studies are needed to clarify this.

Plasma biochemical parameters indicate the body’s metabolism and digestive activity and reflect the host’s health status, mainly affected by diet, environment, and age [25]. For example, Madeira et al. [26] reported that dietary arginine supplementation could increase the plasma total lipids and triacylglycerol levels in commercial crossbred pigs. Similarly, Pardo et al. [27] found that long-term heat stress might increase the plasma urea level in Iberian pigs. In the present study, plasma CHO and LDL-C levels were lower at 80 and 125 days of age while TG, LDL-C, and UN levels were higher at 185 days of age in TB and XB pigs than those in DR pigs, suggesting that the TB and XB pigs have higher fat deposition and lower protein accretion ability than the DR pigs during the finishing period. Xing et al. [28] also found that the creatine kinase mitochondrial 2 (CKMT2) expression related to fat deposition was higher in domestic pigs (Songliao black pigs) than in foreign pigs (Landrace pig). Moreover, Saqui-Salces et al. [29] reported that the higher plasma UN level indicated that the activity of protein catabolism is stronger than synthesis.

In addition to biochemical parameters, plasma free amino acids content reflect the dynamic states of the metabolic flux of amino acids absorbed from the small intestine, as well as being a key index of body protein anabolism and catabolism [30]. In the present study, essential amino acids, such as Met, Thr, Val, Iso, Leu, and Phe, had no significant differences among the three pig breeds, suggesting that the nutritional status of these three pig breeds was similar [31]. In addition, plasma Ala, Asp, Gly, Pro, and Tau contents in the TB and XB pigs were higher at 80 days of age but were lower at 125 and 185 days of age than those in the DR pigs, suggesting that the protein deposition happened in the finishing period in the TB and XB pigs and in the growing period in the DR pig. Ashworth et al. [32] reported that the plasma contents of Ala, Gly, and Asp in the Meishan pig were higher in the early growth period. Liao et al. [33] also reported that several amino acids, including Arg, Orn, Pro, Asp, and Ala, may be degraded by the small intestine mucosa and bacteria in the first pass, resulting in their lower plasma concentrations. Thus, the low plasma amino acids content in the TB and XB pigs during the finishing period in the present study might be related to the faster growth and stronger microbial fermentation in their hindgut.

The capacity of nutrient digestion and absorption in the small intestine is indicated by intestinal morphology and digestive enzyme activities [34]. Intestinal morphology is usually evaluated using the VH, CD, and VH/CD ratio. A higher VH, a lower CD and VH/CD ratio, and a larger absorption area indicate more mature cells, resulting in stronger absorption and secretory capacity of the intestine [35]. In the present study, DR pigs had higher jejunal VH at 185 days of age, while the XB and DR pigs had higher ileal VH at 185 days of age. However, the pig breed and different growth stages did not affect the VH/CD ratio of pigs. These results suggest that the villus status was affected by the pig breed and growth period, which was similar to the results of Rubio et al. [36], who reported that the VH/CD ratio of the small intestine was not different, and the differences in the intestinal morphology between Iberian and lean pigs disappeared with growth.

The digestive enzyme activity reflects not only the intestinal digestive ability but also the intestinal maturation in animals [37]. The activity of disaccharidases, especially invertase, lactase, and maltase, can characterize intestinal functional development and integrity. Decreased activities of these enzymes may reflect damage to the mucosa [38]. In the present study, the XB and TB pigs had higher invertase, lactase, and maltase activities in the jejunum at 80, 125, and 185 days of age, while the XB pigs had higher invertase, lactase, and maltase activities in the ileum at 80 days of age, suggesting that the TB and XB pigs have the stronger digestive ability and better development of the small intestine than the DR pigs. This might be related to the higher intestine index of domestic pigs which demand more energy to maintain the growth of the small intestine, and these disaccharidases can catalyze the carbohydrates and provide energy for intestinal development. Kemp et al. [39] also reported that Meishan pigs had higher digestive enzyme activities compared with the Dutch Landrace pigs.

The ATTD is a key indicator of the digestive ability of animals and is affected by many factors, such as diet and age. In the present study, the ATTD values for DM, ADF, GE, and amino acids of TB and XB pigs were higher at 80 days of age and were lower at 125 and 185 days of age, except for the high ATTD of CP and amino acids in the TB pigs at 185 days of age. These findings suggest that domestic pigs exhibited a higher nutrient digestibility during the growing period. The genetic factor may cause this higher nutrient digestibility with the higher concentration of digestive enzymes from the microbe in the hindgut [40]. Moreover, the lower ATTD of TB and XB pigs during the finishing period may be related to insufficient dietary fiber content for the finishing requirement. Noblet et al. [41] reported that digestive efficiency increases with a high-fiber diet in selecting growing pigs.

The majority of absorptive enterocytes exist in the small intestine, which can express specialized nutrient transporters involved in the transportation and uptake of luminal nutrients, and the expression of these transporters can reflect the absorption ability of the intestine [42]. *GLUT1* and *GLUT2* are responsible for the basic supply of cells with glucose, and *GLUT5* exhibits no glucose transport activity and is responsible for the uptake of fructose [43]. In the present study, the DR and TB pigs had higher expression of *GLUT5* in the jejunum and ileum compared with the XB pigs, suggesting that the DR and TB pigs had stronger fructose transportation capacity than the XB pigs. In addition, *GLUT* and *SGLT* levels differed with age and the intestinal segment (i.e., jejunum and ileum). The *SGLT* and (*GLUT* families are the transporter proteins linked to glucose transportation. Specifically, *SGLT1* is a high-affinity and low-capacity sodium–glucose symporter and is expressed mainly in the small intestine [44]. These results might be related to the different diets at different ages, as well as the different energy needs in the different intestinal segments.

*PEPT1*, *SLC1A1*, *SLC7A7*, and *SLC6A19* are responsible for the uptake of peptides, acidic amino acids, basic amino acids, and neutral amino acids, respectively [45]. The *SLC1A1*, *SLC7A7*, and *SLC6A19* expression levels were slightly or significantly higher in the jejunum and ileum of DR pigs at 80 days of age, in the jejunum of XB pigs at 125 days of age, and in the ileum of TB pigs at 80 and 185 days of age. These results suggest that the expression levels of amino acid transporters are related to the growth period, which might be related to the level of amino acid metabolism. Previously, Yang et al. [46] reported that the expression levels of *SLC6A19* and *SLC1A5* in piglets with low BW were lower in comparison with those of their high-BW littermates, accompanied by the lower contents of plasma, muscle, and liver amino acids during the early suckling period.

*FABP1* and *FABP2* are responsible for the transportation of lipids in the small intestine [47]. Gajda and Storch [48] reported that the expression of *FABP2* in the jejunum was higher. Our findings showed that the XB pigs had higher *FABP1* and *FABP2* expression levels in the jejunum and ileum at 125 days of age, suggesting a stronger fatty acid transportation ability. These results might be related to the higher lipase level in the jejunum of XB pigs at 125 days of age, resulting in higher levels of fatty acids in the small intestine.

## 5. Conclusions

In summary, changes in the growth performance, small intestinal function and morphology, plasma biochemical parameters, and apparent total tract digestibility appeared differently among different pig breeds. The TB and XB pigs exhibited lower growth performance while having higher digestive enzyme activities than the DR pigs during different growth periods. Furthermore, changes were related to the ages of pigs; specifically, differences in feed efficiency were shown in early growth and then disappeared with age. However, the underlying mechanism needs to be further studied. 

## Figures and Tables

**Table 1:** Ingredient composition and nutrient levels of diets, % as dry matter basis.

Ingredient	Pre-Nursey Period (8–20 kg)	Late-Nursey Period (20–35 kg)	Growing Period (35–60 kg)	Finishing Period (60–90 kg)
Corn (8% CP)	36.42	63.08	69.59	64.73
Extruded corn (7.8% CP)	27.00	/	/	/
Extruded soybeans (35.5% CP)	3.00	6.00	/	/
Soybean meal (44% CP)	19.80	16.59	17.14	12.50
Fish meal (62.5% CP)	5.97	1.80	2.30	/
Whey powder (low protein)	4.50	/	/	/
Wheat bran (14.3% CP)	/	9.37	9.00	20.45
Soybean oil	1.00	0.50	/	/
Stone powder	0.70	0.90	0.65	0.80
CaHPO_4_	0.40	0.65	0.35	0.30
NaCl	0.30	0.30	0.30	0.30
L-Lysine hydrochloride (78%)	0.30	0.20	0.15	0.40
DL-Methionine	0.08	0.03	0.03	0.02
L-Threonine (98.5%)	0.10	0.17	0.09	0.10
L-Tryptophan (98%)	0.04	0.02	0.01	0.01
Choline chloride	0.15	0.15	0.15	0.15
Vitamin premix	0.04	0.04	0.04	0.04
Mineral element premix	0.20	0.20	0.20	0.20
Total	100.00	100.00	100.00	100.00
Nutritional level				
Digestive energy (MJ/kg)	14.19	13.73	13.48	12.98
Crude protein (CP)	19.11	17.27	16.05	14.04
Lysine	1.22	0.93	0.81	0.84
Methionine	0.39	0.28	0.26	0.20
Methionine + cysteine	0.66	0.55	0.52	0.44
Threonine	0.76	0.70	0.56	0.49
Tryptophan	0.24	0.19	0.17	0.15
Calcium	0.81	0.76	0.58	0.53
Available phosphorus	0.42	0.34	0.28	0.22

Each kg of vitamin premix contained VA, 1800 IU; VD_3_, 200 IU; VE, 11 mg; VK, 0.5 mg; VB_1_, 1 mg; VB_2_, 3.5 mg; VB_6_, 1.5 mg; VB_12_, 17.5 mg; niacin, 15 mg; pantothenic acid, 10 mg; folate, 0.3 mg; and biotin, 0.05 mg during 8−20 kg body weight (BW); VA, 5000 IU; VC, 20 mg; VE, 50 mg; VK, 1 mg; VB_1_, 2 mg; VB_2_, 10 mg; VB_6_, 4 mg; VB_12_, 0.03 mg; niacin, 30 mg; pantothenic acid, 20 mg; folate, 0.6 mg; and biotin, 0.3 mg during 20−35 kg BW; VA, 2250 IU; VD_3_, 220 IU; VE, 16 mg; VK, 0.5 mg; VB_1_, 2 mg; VB_2_, 5 mg; VB_6_, 4 mg; VB_12_, 0.03 mg; niacin, 30 mg; pantothenic acid, 20 mg; folate, 0.3 mg; and biotin, 0.2 mg during 35−60 kg and 60−90 kg BW, respectively. Per kg of mineral premix containing Fe, 105 mg; Cu, 6 mg; Zn, 100 mg; Mn, 4 mg; I, 0.14 mg; and Se, 0.3 mg during 8−20 kg BW; Fe, 180 mg; Cu, 12 mg; Zn, 150 mg; Mn, 3 mg; I, 0.14 mg; and Se, 0.25 mg during 20−35 kg BW; Fe, 100 mg; Cu, 10 mg; Zn,100 mg; Mn, 2 mg; I, 0.14 mg; and Se, 0.25 mg during 35−60 kg BW; and Fe, 80 mg; Cu, 5 mg; Zn, 80 mg; Mn, 3 mg; I, 0,14 mg; and Se, 0.25 mg during 60−90 kg BW, respectively.

**Table 2:** Primer sequences of the target genes.

Target Gene	Accession No.	Primer Sequences (5′–3′)	Product Size (bp)
*FABP1*	NM_001004046.2	F: TGAACTCAACGGTGACATA	75
		R: ATTCTCTTGCTGATTCTCTTG	
*FABP2*	NM_001031780.1	F: CAGCCTCGCAGACGGAACTGAA	217
		R: GTGTTCTGGGCTGTGCTCCAAGA	
*FABP4*	NM_001002817.1	F: TGGAAACTTGTCTCCAGTG	147
		R: GGTACTTTCTGATCTAATGGTG	
*GLUT1*	XM_003482115.1	F: TGCTCATCAACCGCAATGA	72
		R: GTTCCGCGCAGCTTCTTC	
*GLUT2*	NM_001097417.1	F: CCAGGCCCCATCCCCTGGTT	96
		R: GCGGGTCCAGTTGCTGAATGC	
*GLUT5*	XM_021095282.1	F: CCCAGGAGCCGGTCAAG	60
		R: TCAGCGTCGCCAAAGCA	
*PEPT1*	NM_214347.1	F: GGATAGCCTGTACCCCAAGCT	73
		R: CATCCTCCACGTGCTTCTTGA	
*SGLT1*	NM_001164021.1	F: GGCTGGACGAAGTATGGTG	153
		R: ACAACCACCCAAATCAGAGC	
*SLC1A1*	NM_001164649.1	F: GGCACCGCACTCTACGAAGCA	177
		R: GCCCACGGCACTTAGCACGA	
*SLC1A5*		F: GATTGTGGAGATGGAGGATGTGG	128
		R: TGCGAGTGAAGAGGAAGTAGATGAGA	
*SLC6A19*	XM_003359855.4	F: TCTGTCCACAACAACTGCGAG	206
		R: CAGCGAAGTTCTCCTGCGTC	
*SLC7A7*	NM_001110421.1	F: TCAAGTGGGGAACCCTGGTA	259
		R: ATGGAGAGGGGCAGATTCCT	
*SLC7A9*		F: GAACCCAAGACCACAAATC	180
		R: ACCCAGTGTCGCAAGAAT	
*SLC15A1*		F: GGATAGCCTGTACCCCAAGCT	73
		R: CATCCTCCACGTGCTTCTTGA	

*FABP*, fatty acid binding protein; *GLUT*, glucose transporter; *PEPT1*, peptide transporter 1; *SGLT1*, sodium–glucose linked transporter 1; *SLC1A1*, solute carrier family 1 member 1; *SLC1A5*, solute carrier family 1 member 5; *SLC6A19*, solute carrier family 6 member 19; *SLC7A7*, solute carrier family 7 member 7; *SLC7A9*, solute carrier family 7 member 9; *SLC15A1*, solute carrier family 15 member 1.

**Table 3:** Growth performance and feed efficiency of three pig breeds at different ages.

Item	TB Pig	XB Pig	DR Pig	SEM	*p* Value
35–80 days of age					
Initial weight (kg)	6.68 ^b^	5.63 ^b^	10.18 ^a^	0.48	<0.001
ADG (kg/day)	0.11 ^b^	0.10 ^b^	0.44 ^a^	0.03	<0.001
ADFI (kg/day)	0.26 ^b^	0.17 ^c^	1.03 ^a^	0.08	<0.001
FCR	2.53 ^a^	1.61 ^b^	2.40 ^a^	0.12	0.002
Final weight (kg)	11.38 ^b^	9.42 ^b^	22.94 ^a^	1.26	<0.001
80–125 days of age					
Initial weight (kg)	10.46 ^b^	10.66 ^b^	24.93 ^a^	1.42	<0.001
ADG (kg/day)	0.54 ^b^	0.50 ^b^	0.81 ^a^	0.03	<0.001
ADFI (kg/day)	1.21 ^b^	1.15 ^b^	1.89 ^a^	0.08	<0.001
FCR	2.24	2.33	2.36	0.05	0.626
Final weight (kg)	34.79 ^b^	33.23 ^b^	61.33 ^a^	2.81	<0.001
125–185 days of age					
Initial weight (kg)	37.92 ^b^	36.77 ^b^	58.47 ^a^	2.13	<0.001
ADG (kg/day)	0.66 ^b^	0.60 ^b^	0.77 ^a^	0.02	<0.001
ADFI (kg/day)	2.62 ^b^	2.60 ^b^	3.46 ^a^	0.09	<0.001
FCR	4.10	4.40	4.52	0.10	0.262
Final weight (kg)	77.08 ^b^	72.60 ^b^	104.65 ^a^	3.03	<0.001

Data are presented as means, SEM, and *p* values (*n* = 10). Values in the same row without a common superscript letter are different (*p* < 0.05). TB pig, Taoyuan black pig; XB pig, Xiangcun black pig; DR pig, Duroc pig; ADG, average daily gain; ADFI, average daily feed intake; FCR, feed/gain ratio.

**Table 4:** Plasma biochemical parameters of three pig breeds at different ages.

Item	TB Pig	XB Pig	DR Pig	SEM	*p* Value
80 days of age					
ALB (g/L)	43.49	45.90	41.11	1.40	0.366
ALT (U/L)	55.49	50.84	60.81	2.95	0.367
AMS (U/L)	2609.13	2351.30	2667.00	104.28	0.417
AST (g/L)	147.57 ^a^	125.25 ^ab^	78.30 ^b^	11.66	0.034
CHE (mmol/L)	759.29	729.60	800.20	30.72	0.629
CHO (mmol/L)	2.24 ^b^	2.37 ^b^	3.25 ^a^	0.14	0.002
GLU (mmol/L)	6.24	5.87	5.89	0.18	0.688
HDL-C (mmol/L)	1.04	1.11	1.11	0.05	0.784
LDH (U/L)	1142.29	912.80	1078.40	49.92	0.159
LDL-C (mmol/L)	1.35 ^b^	1.49 ^b^	2.45 ^a^	0.12	<0.001
LIP (U/L)	28.57 ^a^	30.35 ^a^	19.52 ^b^	1.41	0.001
NH_3_ (μmol/L)	261.51	227.51	178.49	16.08	0.129
TG (mmol/L)	0.45	0.42	0.57	0.03	0.068
TP (g/L)	64.86	69.12	71.49	2.04	0.443
UN (U/L)	3.50	3.52	3.08	0.17	0.497
125 days of age					
ALB (g/L)	60.85	55.41	60.53	8.26	0.313
ALT (U/L)	55.40	43.23	57.26	14.76	0.108
AMS (U/L)	2727.25	2384.75	2676.78	453.6	0.271
AST (g/L)	46.63	48.71	55.89	10.14	0.141
CHE (mmol/L)	767.38	727.56	781.67	137.37	0.706
CHO (mmol/L)	2.67 ^b^	2.59 ^b^	3.38 ^a^	0.54	0.001
GLU (mmol/L)	7.73	7.66	6.58	1.47	0.188
HDL-C (mmol/L)	1.13 ^b^	0.92 ^c^	1.34 ^a^	0.24	<0.001
LDH (U/L)	661.13	733.88	754.33	110.47	0.201
LDL-C (mmol/L)	1.77 ^b^	1.90 ^b^	2.33 ^a^	0.39	0.003
LIP (U/L)	31.53 ^a^	10.59 ^b^	9.09 ^b^	11.62	<0.001
NH_3_ (μmol/L)	151.07 ^b^	170.53 ^b^	224.92 ^a^	51.89	0.006
TG (mmol/L)	0.47	0.37	0.44	0.12	0.353
TP (g/L)	78.13	80.01	89.10	10.55	0.061
UN (U/L)	4.45	3.45	3.84	1.35	0.345
185 days of age					
ALB (g/L)	61.81	59.08	66.82	1.85	0.227
ALT (U/L)	90.41 ^a^	83.14 ^a^	55.56 ^b^	5.17	0.007
AMS (U/L)	2649.33	2566.56	2674.78	85.71	0.874
AST (g/L)	124.13 ^a^	91.00 ^ab^	60.00 ^b^	9.25	0.010
CHE (mmol/L)	876.33	695.67	740.78	32.94	0.060
CHO (mmol/L)	3.40	3.12	2.82	0.16	0.332
GLU (mmol/L)	7.19	6.34	6.80	0.24	0.367
HDL-C (mmol/L)	1.02	1.07	1.29	0.06	0.097
LDH (U/L)	1260.63 ^a^	896.13 ^b^	652.33 ^b^	66.54	<0.001
LDL-C (mmol/L)	2.69 ^a^	2.31 ^ab^	1.85 ^b^	0.14	0.041
LIP (U/L)	34.43 ^a^	21.78 ^b^	8.43 ^c^	2.19	<0.001
NH_3_ (μmol/L)	168.41 ^a^	125.08 ^b^	114.67 ^b^	8.20	0.010
TG (mmol/L)	0.59 ^a^	0.46 ^ab^	0.43 ^b^	0.03	0.044
TP (g/L)	89.24	89.44	88.14	2.55	0.977
UN (U/L)	6.13 ^a^	5.22 ^a^	3.59 ^b^	0.32	0.001

Data are presented as means, SEM, and *p* values (*n* = 10). Values in the same row without a common superscript letter are different (*p* < 0.05). TB pig, Taoyuan black pig; XB pig, Xiangcun black pig; DR pig, Duroc pig; ALB, albumin; ALT, alanine aminotransferase; AMS, amylase; AST, aspartate aminotransferase; CHE, cholinesterase; CHO, cholesterol; GLU, glucose; HDL-C, high-density lipoprotein-cholesterol; LDH, lactate dehydrogenase; LDL-C, low-density lipoprotein-cholesterol; LIP, lipase; NH_3_, ammonia; TG, triglyceride; TP, total protein, UN, urea nitrogen.

**Table 5:** Plasma free amino acids composition of three pig breeds at 80 days of age (μg/mL).

Item	TB Pig	XB Pig	DR Pig	SEM	*p* Value
1-MH	1.93	2.02	4.41	1.09	0.575
3-MH	1.28	1.19	1.12	0.06	0.696
α-AAA	5.52 ^a^	3.11 ^b^	3.69 ^b^	0.33	0.005
α-ABA	0.73 ^b^	1.26 ^b^	2.00 ^a^	0.14	<0.001
Ala	44.73 ^a^	47.34 ^a^	23.84 ^b^	2.55	<0.001
Arg	18.00 ^a^	14.16 ^b^	14.12 ^b^	0.64	0.018
Asp	2.37 ^a^	2.09 ^a^	1.28 ^b^	0.13	<0.001
β-Ala	2.01	1.87	2.61	0.14	0.065
Car	8.18 ^a^	5.46 ^b^	2.77 ^c^	0.55	<0.001
Cit	11.13	11.74	9.88	0.36	0.086
Cys	9.26	8.74	9.17	0.24	0.653
Cysthi	0.76	1.2	0.93	0.08	0.098
ETA	2.00 ^ab^	1.60 ^b^	2.64 ^a^	0.14	0.002
Glu	35.90 ^a^	33.41 ^a^	24.08 ^b^	1.79	0.011
Gly	105.75 ^a^	120.39 ^a^	69.12 ^b^	6.34	<0.001
His	1.33 ^b^	0.84 ^b^	2.67 ^a^	0.23	<0.001
Hylys	1.69 ^a^	0.72 ^b^	0.52 ^b^	0.19	0.019
HYP	18.1	15.07	14.99	0.70	0.188
Ile	9.88	10.88	11.17	0.33	0.291
Leu	13.89 ^b^	16.49 ^ab^	17.21 ^a^	0.55	0.037
Lys	16.68 ^b^	24.63 ^a^	17.72 ^b^	1.18	0.007
Met	5.54	4.26	4.86	0.23	0.108
Orn	0.13	0.21	0.15	0.02	0.391
Phe	7.29 ^b^	10.27 ^a^	10.68 ^a^	0.37	<0.001
Pro	21.36 ^a^	24.05 ^a^	16.51 ^b^	20.59	<0.001
P-Ser	6.39	7.29	5.48	0.41	0.182
Sar	1.46 ^b^	3.71 ^a^	0.70 ^c^	0.32	<0.001
Ser	12.65 ^b^	18.37 ^a^	10.03 ^b^	0.96	<0.001
Tau	11.92 ^a^	11.21 ^ab^	7.86 ^b^	0.70	0.032
Thr	3.79 ^b^	4.79 ^b^	8.22 ^a^	0.49	<0.001
Tyr	7.59 ^c^	9.61 ^b^	11.59 ^a^	0.43	<0.001
Val	13.23 ^b^	16.97 ^a^	18.09 ^a^	0.71	0.012

Data are presented as means, SEM, and *p* values (*n* = 10). Values in the same row without a common superscript letter are different (*p* < 0.05). TB pig, Taoyuan black pig; XB pig, Xiangcun black pig; DR pig, Duroc pig; α-AAA, alpha-aminoadipic acid; α-ABA, alpha-aminobutyric acid; Cit, citrulline; Ala, alanine; Arg, arginine; Asp, aspartic acid; Cys, cysteine; Cysthi, cystathionine; β-Ala, beta-alanine; ETA, ethanolamine; 1-MH, 1-methylhistidine; 3-MH, 3-methylhistidine; Orn, L-ornithine; HYP, hydroxyproline; Glu, glutamic acid; Gly, glycine; His, histidine; Ile, isoleucine; Leu, leucine; Lys, lysine; Met, methionine; Phe, phenylalanine; Pro, proline; Sar, sarcosine; Ser, serine; Thr, threonine; Tyr, tyrosine; Val, valine. The tables below are the same.

**Table 6:** Plasma free amino acids composition of three pig breeds at 125 days of age (μg/mL).

Item	TB Pig	XB Pig	DR Pig	SEM	*p* Value
1-MH	1.63	6.53	6.17	2.24	0.709
3-MH	1.11	1.09	1.34	0.08	0.396
α-AAA	7.22 ^a^	6.02 ^ab^	3.82 ^b^	0.50	0.010
α-ABA	1.18	1.47	1.68	0.14	0.332
Ala	24.19 ^b^	35.49 ^a^	28.96 ^ab^	1.57	0.014
Arg	17.25	13.88	18.30	0.94	0.134
Asp	0.86 ^b^	1.76 ^a^	1.40 ^a^	0.12	0.005
β-Ala	1.54 ^b^	1.88 ^ab^	2.27 ^a^	0.13	0.046
Car	4.31	4.77	3.25	0.29	0.064
Cit	10.91	8.82	7.51	0.68	0.106
Cys	8.81	9.05	8.41	0.37	0.776
Cysthi	1.10	1.09	1.08	0.07	0.992
ETA	3.97 ^a^	1.83 ^b^	0.48 ^c^	0.40	<0.001
Glu	17.22 ^b^	31.68 ^a^	21.48 ^b^	1.60	<0.001
Gly	95.23 ^a^	78.78 ^ab^	64.82 ^b^	4.03	0.002
His	1.12	1.66	-	0.21	0.211
Hylys	-	2.25	2.47	0.14	0.469
HYP	9.58	11.07	11.26	10.75	0.648
Ile	16.17	13.43	16.34	0.77	0.233
Leu	26.02	22.50	28.38	1.25	0.142
Lys	24.85	22.30	24.96	1.17	0.637
Met	4.23	3.91	4.96	0.24	0.192
Orn	3.02	3.53	5.19	0.63	0.328
Phe	11.99	12.58	14.06	0.48	0.179
Pro	26.78	25.39	21.63	24.39	0.200
P-Ser	4.95 ^a^	2.03 ^b^	3.88 ^a^	0.32	<0.001
Sar	-	1.11	1.48	0.20	0.392
Ser	11.68	12.79	11.11	0.58	0.493
Tau	6.06 ^b^	7.16 ^b^	9.86 ^a^	0.56	0.010
Thr	17.12	16.19	14.66	0.73	0.377
Tyr	14.59	13.40	16.54	0.68	0.156
Val	29.73	25.11	30.38	1.51	0.318

Values in the same row without a common superscript letter are different (*p* < 0.05).

**Table 7:** Plasma free amino acids composition of three pig breeds at 185 days of age (μg/mL).

Item	TB Pig	XB Pig	DR Pig	SEM	*p* Value
1-MH	-	1.51	3.74	0.59	0.070
3-MH	2.01 ^a^	1.81 ^ab^	1.60 ^b^	0.07	0.037
α-AAA	5.06 ^b^	5.35 ^b^	8.36 ^a^	0.56	0.021
α-ABA	3.16 ^a^	2.40 ^a^	1.26 ^b^	0.25	0.002
Ala	24.22 ^b^	25.23 ^b^	33.93 ^a^	1.54	0.011
Arg	28.79 ^a^	16.45 ^b^	19.44 ^b^	1.34	<0.001
Asp	1.05	0.82	0.90	0.07	0.458
β-Ala	1.35 ^a^	0.64 ^b^	0.76 ^b^	0.08	<0.001
Car	4.94	5.37	5.90	0.34	0.530
Cit	10.21	11.22	9.82	0.45	0.456
Cys	9.01	8.59	8.89	0.25	0.820
Cysthi	1.11	1.15	1.15	0.06	0.959
ETA	0.33 ^b^	0.28 ^b^	0.50 ^a^	0.03	0.001
Glu	15.19	15.61	18.77	0.73	0.097
Gly	39.11 ^b^	47.06 ^b^	71.66 ^a^	3.44	<0.001
His	-	1.04	2.38	0.39	0.084
Hylys	1.73 ^a^	1.23 ^b^	1.87 ^a^	0.09	0.008
HYP	5.74 ^b^	5.98 ^b^	10.24 ^a^	0.57	<0.001
Ile	19.00	14.04	15.20	0.89	0.050
Leu	29.25	22.82	27.13	1.15	0.060
Lys	26.39	23.12	28.28	0.90	0.054
Met	5.59 ^ab^	4.81 ^b^	5.81 ^a^	0.17	0.042
Phe	16.53 ^a^	13.15 ^b^	13.71 ^b^	0.47	0.004
Pro	16.09 ^b^	16.09 ^b^	20.94 ^a^	0.60	<0.001
P-Ser	2.14	1.84	1.82	0.14	0.619
Sar	1.15 ^b^	1.33 ^b^	2.13 ^a^	0.16	0.029
Ser	9.79 ^ab^	8.68 ^b^	11.14 ^a^	0.34	0.007
Tau	13.61	12.30	12.01	0.54	0.456
Thr	19.62	16.44	18.06	0.55	0.054
Tyr	17.14 ^a^	13.41 ^b^	17.72 ^a^	0.53	<0.001
Val	8.95 ^c^	20.89 ^b^	32.97 ^a^	2.67	<0.001

Values in the same row without a common superscript letter are different (*p* < 0.05).

**Table 8:** Jejunal morphology of three pig breeds at different ages.

Item	TB Pig	XB Pig	DR Pig	SEM	*p* Value
80 days of age					
VH (μm)	363.95 ^ab^	416.49 ^a^	329.92 ^b^	12.70	0.028
CD (μm)	220.69	267.54	235.44	9.01	0.119
VH/CD	1.72	1.57	1.43	0.08	0.370
125 days of age					
VH (μm)	430.40	436.42	379.90	12.69	0.135
CD (μm)	246.89	238.49	229.44	6.87	0.613
VH/CD	1.77	1.89	1.69	0.04	0.183
185 days of age					
VH (μm)	394.63 ^b^	371.60 ^b^	506.25 ^a^	20.64	0.001
CD (μm)	231.56	221.44	270.18	9.96	0.082
VH/CD	1.76	1.75	1.95	0.05	0.193

Data are presented as means, SEM, and *p* values (*n* = 10). Values in the same row without a common superscript letter are different (*p* < 0.05). TB pig, Taoyuan black pig; XB pig, Xiangcun black pig; DR pig, Duroc pig; VH, villus height; CD, crypt depth; VH/CD, villus height/crypt depth ratio. The table below is the same.

**Table 9:** Ileal morphology of three pig breeds at different ages.

Item	TB Pig	XB Pig	DR Pig	SEM	*p* Value
80 days of age					
VH (μm)	312.86	327.53	354.45	8.14	0.101
CD (μm)	161.82 ^b^	168.19 ^b^	215.74 ^a^	7.56	0.001
VH/CD	1.99	1.98	1.69	0.07	0.135
125 days of age					
VH (μm)	428.77 ^a^	384.87 ^ab^	321.92 ^b^	16.52	0.019
CD (μm)	184.39	184.98	178.53	6.89	0.933
VH/CD	2.38	2.19	1.86	0.10	0.114
185 days of age					
VH (μm)	329.71 ^b^	463.84 ^a^	471.46 ^a^	21.75	0.005
CD (μm)	150.84	168.13	179.72	5.53	0.110
VH/CD	2.27	2.84	2.73	0.13	0.252

Values in the same row without a common superscript letter are different (*p* < 0.05).

**Table 10:** Jejunal digestive enzyme activities of three pig breeds at different ages (U/g).

Item	TB Pig	XB Pig	DR Pig	SEM	*p* Value
80 days of age					
Amylase	4.75 ^b^	7.36 ^a^	8.38 ^a^	0.52	0.011
Chymase	11.02 ^b^	29.12 ^a^	5.67 ^b^	2.60	<0.001
Invertase	32.99 ^a^	34.30 ^a^	17.79 ^b^	2.28	0.001
Lactase	19.05 ^a^	18.46 ^a^	9.77 ^b^	1.06	<0.001
Lipase	2.56 ^b^	3.68 ^a^	2.16 ^b^	0.21	0.003
Maltase	31.47 ^a^	29.40 ^a^	13.07 ^b^	1.88	<0.001
Trypsin	12.32 ^a^	11.22 ^a^	7.65 ^b^	0.57	<0.001
125 days of age					
Amylase	3.93 ^b^	10.18 ^a^	8.27 ^a^	0.73	<0.001
Chymase	6.64 ^a^	3.49 ^b^	6.81 ^a^	0.46	<0.001
Invertase	22.48 ^a^	20.37 ^ab^	14.62 ^b^	1.18	0.041
Lactase	14.42 ^a^	10.01 ^b^	9.07 ^b^	0.61	<0.001
Lipase	1.03 ^b^	2.12 ^a^	2.11 ^a^	0.18	0.014
Maltase	25.81 ^a^	15.22 ^b^	13.53 ^b^	1.31	<0.001
Trypsin	8.38	8.02	8.56	0.28	0.747
185 days of age					
Amylase	13.72	13.14	10.29	0.70	0.091
Chymase	18.17 ^a^	13.69 ^b^	12.73 ^b^	0.87	0.021
Invertase	31.78	26.13	22.96	1.63	0.085
Lactase	23.65 ^a^	19.51 ^ab^	15.87 ^b^	1.22	0.028
Lipase	5.35 ^a^	4.40 ^ab^	3.60 ^b^	0.25	0.012
Maltase	16.55	12.86	11.84	0.84	0.055
Trypsin	13.95 ^a^	10.96 ^ab^	9.45 ^b^	0.68	0.018

Data are presented as means, SEM, and *p* values (*n* = 8). Values in the same row without a common superscript letter are different (*p* < 0.05). TB pig, Taoyuan black pig; XB pig, Xiangcun black pig; DR pig, Duroc pig. The table below is the same.

**Table 11:** Ileal digestive enzyme activities of three pig breeds at different ages (U/g).

Item	TB Pig	XB Pig	DR Pig	SEM	*p* Value
80 days of age					
Amylase	3.54 ^b^	9.24 ^a^	7.07 ^a^	0.71	<0.001
Chymase	7.77 ^b^	17.71 ^a^	12.03 ^b^	1.27	<0.001
Invertase	21.68 ^b^	52.25 ^a^	19.78 ^b^	3.65	<0.001
Lactase	24.79 ^b^	42.92 ^a^	23.86 ^b^	2.55	<0.001
Lipase	3.11 ^b^	7.33 ^a^	2.98 ^b^	0.57	<0.001
Maltase	29.22 ^b^	66.46 ^a^	26.60 ^b^	4.59	<0.001
Trypsin	9.92 ^c^	24.80 ^a^	15.03 ^b^	1.60	<0.001
125 days of age					
Amylase	13.94	12.60	12.57	0.48	0.429
Chymase	19.24	16.14	16.78	0.62	0.092
Invertase	30.69	26.29	25.85	1.02	0.097
Lactase	21.57	18.75	19.28	0.74	0.263
Lipase	4.50	3.89	4.06	0.15	0.222
Maltase	16.10	13.48	13.85	0.56	0.115
Trypsin	11.20	9.51	9.68	0.38	0.126
185 days of age					
Amylase	12.19	13.75	11.81	0.42	0.129
Chymase	17.17	18.53	15.00	0.80	0.192
Invertase	28.87	32.68	25.78	1.32	0.086
Lactase	21.20	22.57	18.66	0.84	0.144
Lipase	4.54	4.76	3.90	0.20	0.186
Maltase	14.62	15.75	13.26	0.65	0.293
Trypsin	12.49	13.08	9.71	0.65	0.064

Values in the same row without a common superscript letter are different (*p* < 0.05).

**Table 12:** Apparent total tract digestibility of three pig breeds at 80 days of age (%).

Item	TB Pig	XB Pig	DR Pig	SEM	*p* Value
ADF	85.38 ^a^	85.78 ^a^	81.60 ^b^	0.75	0.029
Ash	43.64	41.92	37.90	2.15	0.556
CP	79.69	74.03	73.55	1.23	0.090
DM	84.04 ^a^	82.78 ^a^	78.21 ^b^	0.83	0.006
EE	84.30	82.97	86.77	1.32	0.482
GE	84.56 ^a^	83.12 ^ab^	80.08 ^b^	0.76	0.046
NDF	84.77	82.76	80.23	0.8	0.068
Ala	77.11	73.14	73.48	1.33	0.449
Cys	97.41	97.25	97.91	0.39	0.771
Gly	81.11	78.54	78.12	1.02	0.478
Ile	84.13	81.39	81.56	0.94	0.455
Leu	73.67	68.57	67.73	1.58	0.290
Met	98.34	98.01	98.07	3.51	0.417
Phe	84.28	81.50	80.40	0.91	0.227
Pro	83.20	80.29	80.31	0.98	0.421
Ser	84.66	82.51	80.40	0.79	0.097
Thr	84.14 ^a^	82.16 ^ab^	79.18 ^b^	0.84	0.049
Tyr	94.13 ^a^	92.90 ^a^	89.99 ^b^	1.19	<0.001
Val	79.55	76.14	76.25	1.19	0.460
Asp	68.69	62.89	60.97	1.86	0.245
Glu	59.83	51.89	50.65	2.46	0.293
Arg	90.66 ^a^	88.50 ^a^	83.91 ^b^	0.81	0.001
His	95.18 ^a^	94.14 ^a^	92.07 ^b^	0.38	0.001
Lys	78.69	74.75	73.66	1.29	0.284

Data are presented as means, SEM, and *p* values (*n* = 10). Values in the same row without a common superscript letter are different (*p* < 0.05). TB pig, Taoyuan black pig; XB pig, Xiangcun black pig; DR pig, Duroc pig; ADF, acid detergent fiber; Ash, crude ash; CP, crude protein; DM, dry matter; EE, ether extract; GE, gross energy; NDF, neutral detergent fiber; Ala, alanine; Cys, cysteine; Gly, glycine; Ile, isoleucine; Leu, leucine; Met, methionine; Phe, phenylalanine; Pro, proline; Ser, serine; Thr, threonine; Tyr, tyrosine; Val, valine; Asp, aspartic acid; Glu, glutamic acid; Arg, arginine; His, histidine; Lys, lysine. The tables below are the same.

**Table 13:** Apparent total tract digestibility of three pig breeds at 125 days of age (%).

Item	TB Pig	XB Pig	DR Pig	SEM	*p* Value
ADF	80.67 ^b^	80.52 ^b^	83.91 ^a^	0.59	0.020
Ash	38.71	35.63	38.95	1.52	0.620
CP	72.46 ^b^	70.77 ^b^	79.66 ^a^	1.14	0.001
DM	75.21 ^ab^	73.72 ^b^	78.67 ^a^	0.73	0.010
EE	87.27	86.97	84.61	0.78	0.314
GE	76.65 ^b^	74.95 ^b^	80.08 ^a^	0.72	0.005
NDF	77.78 ^ab^	75.95 ^b^	80.93 ^a^	0.74	0.011
Ala	67.08 ^b^	67.66 ^b^	76.30 ^a^	1.57	0.019
Cys	97.87 ^b^	97.97 ^b^	98.44 ^a^	0.09	0.020
Gly	75.76 ^b^	76.03 ^b^	82.81 ^a^	1.14	0.010
Ile	78.60 ^b^	78.46 ^b^	84.82 ^a^	1.06	0.012
Leu	61.20 ^b^	61.63 ^b^	71.19 ^a^	1.8	0.029
Met	98.98	99.70	99.54	0.16	0.167
Phe	77.37 ^b^	77.45 ^b^	83.15 ^a^	1.02	0.020
Pro	77.18 ^b^	78.02 ^b^	83.09 ^a^	1.02	0.030
Ser	78.97	79.19	83.67	1.00	0.088
Thr	75.94 ^b^	76.31 ^b^	82.10 ^a^	1.12	0.032
Tyr	87.27 ^b^	87.71 ^b^	90.90 ^a^	1.32	0.013
Val	73.10 ^b^	73.00 ^b^	80.76 ^a^	1.32	0.014
Asp	53.17 ^b^	53.56 ^b^	68.96 ^a^	2.34	0.003
Glu	36.97 ^b^	38.04 ^b^	55.11 ^a^	3.19	0.024
Arg	83.39	83.29	87.61	0.88	0.065
His	90.59 ^b^	90.71 ^b^	93.69 ^a^	0.51	0.014
Lys	67.37 ^b^	67.36 ^b^	77.51 ^a^	1.72	0.013

Values in the same row without a common superscript letter are different (*p* < 0.05).

**Table 14:** Apparent total tract digestibility of three pig breeds at 185 days of age (%).

Item	TB Pig	XB Pig	DR Pig	SEM	*p* Value
ADF	79.71 ^b^	77.15 ^c^	84.36 ^a^	0.69	<0.001
Ash	29.04	27.90	30.98	1.01	0.483
CP	73.80 ^a^	64.56 ^b^	71.12 ^ab^	1.43	0.018
DM	75.44 ^b^	72.83 ^c^	78.36 ^a^	0.52	<0.001
EE	80.07 ^b^	82.89 ^b^	85.77 ^a^	0.93	0.028
GE	75.87 ^b^	73.69 ^c^	79.71 ^a^	0.56	<0.001
NDF	77.52 ^a^	74.04 ^b^	77.37 ^a^	0.53	0.005
Ala	74.36 ^a^	65.21 ^b^	72.87 ^a^	1.29	0.004
Cys	98.63 ^a^	98.01 ^ab^	98.42 ^b^	0.10	0.020
Gly	80.55 ^a^	73.81 ^b^	80.63 ^a^	1.01	0.003
Ile	83.76 ^a^	77.44 ^b^	83.08 ^a^	0.93	0.005
Leu	70.58 ^a^	59.81 ^b^	69.14 ^a^	1.56	0.005
Met	99.37 ^a^	98.19 ^b^	98.48 ^a^	0.19	0.028
Phe	81.77 ^a^	76.34 ^b^	81.61 ^a^	0.85	0.007
Pro	82.20 ^a^	75.80 ^b^	82.26 ^a^	0.94	0.002
Ser	83.10 ^a^	76.55 ^b^	81.90 ^a^	0.90	0.003
Thr	81.36 ^a^	75.16 ^b^	79.94 ^a^	0.84	0.003
Tyr	90.05 ^a^	87.21 ^b^	89.78 ^a^	1.15	0.001
Val	78.95 ^a^	71.19 ^b^	78.25 ^a^	1.15	0.005
Asp	64.42 ^a^	50.74 ^b^	63.69 ^a^	1.93	0.002
Glu	53.12 ^a^	33.85 ^b^	51.21 ^a^	2.60	0.001
Arg	88.32 ^a^	82.85 ^b^	87.02 ^a^	0.67	<0.001
His	93.46 ^a^	90.48 ^b^	92.95 ^a^	0.37	<0.001
Lys	75.71 ^a^	65.96 ^b^	74.74 ^a^	1.31	0.001

Values in the same row without a common superscript letter are different (*p* < 0.05).

## Data Availability

Not applicable.
